# Temporal and Emotional Variations in People’s Perceptions of Mass Epidemic Infectious Disease After the COVID-19 Pandemic Using Influenza A as an Example: Topic Modeling and Sentiment Analysis Based on Weibo Data

**DOI:** 10.2196/49300

**Published:** 2023-11-02

**Authors:** Jing Dai, Fang Lyu, Lin Yu, Yunyu He

**Affiliations:** 1 Kunming University of Science and Technology Kunming China; 2 The First People’s Hospital of Yunnan Province Kunimg China

**Keywords:** mass epidemic infections, sentiment analysis, text mining, spatial differences, temporal differences, influenza A, COVID-19

## Abstract

**Background:**

The COVID-19 pandemic has had profound impacts on society, including public health, the economy, daily life, and social interactions. Social distancing measures, travel restrictions, and the influx of pandemic-related information on social media have all led to a significant shift in how individuals perceive and respond to health crises. In this context, there is a growing awareness of the role that social media platforms such as Weibo, among the largest and most influential social media sites in China, play in shaping public sentiment and influencing people’s behavior during public health emergencies.

**Objective:**

This study aims to gain a comprehensive understanding of the sociospatial impact of mass epidemic infectious disease by analyzing the spatiotemporal variations and emotional orientations of the public after the COVID-19 pandemic. We use the outbreak of influenza A after the COVID-19 pandemic as a case study. Through temporal and spatial analyses, we aim to uncover specific variations in the attention and emotional orientations of people living in different provinces in China regarding influenza A. We sought to understand the societal impact of large-scale infectious diseases and the public’s stance after the COVID-19 pandemic to improve public health policies and communication strategies.

**Methods:**

We selected Weibo as the data source and collected all influenza A–related Weibo posts from November 1, 2022, to March 31, 2023. These data included user names, geographic locations, posting times, content, repost counts, comments, likes, user types, and more. Subsequently, we used latent Dirichlet allocation topic modeling to analyze the public’s focus as well as the bidirectional long short-term memory model to conduct emotional analysis. We further classified the focus areas and emotional orientations of different regions.

**Results:**

The research findings indicate that, compared with China’s western provinces, the eastern provinces exhibited a higher volume of Weibo posts, demonstrating a greater interest in influenza A. Moreover, inland provinces displayed elevated levels of concern compared with coastal regions. In addition, female users of Weibo exhibited a higher level of engagement than male users, with regular users comprising the majority of user types. The public’s focus was categorized into 23 main themes, with the overall emotional sentiment predominantly leaning toward negativity (making up 7562 out of 9111 [83%] sentiments).

**Conclusions:**

The results of this study underscore the profound societal impact of the COVID-19 pandemic. People tend to be pessimistic toward new large-scale infectious diseases, and disparities exist in the levels of concern and emotional sentiments across different regions. This reflects diverse societal responses to health crises. By gaining an in-depth understanding of the public’s attitudes and focal points regarding these infectious diseases, governments and decision makers can better formulate policies and action plans to cater to the specific needs of different regions and enhance public health awareness.

## Introduction

### Background

Over the past century, COVID-19 has emerged as one of the most widespread and impactful diseases. During the COVID-19 pandemic, the rapid transmission and extensive reach of the novel coronavirus, as well as the potentially fatal symptoms of the disease, were a cause for great concern. This not only had a profound impact on people’s lives and economies but also triggered widespread panic, which could lead individuals to experience fear, anxiety, and panic-driven behaviors, such as hoarding supplies or avoiding public places. Therefore, effective risk communication and emotional management are of paramount importance in mitigating the panic effect. In the backdrop of the COVID-19 pandemic, studying people’s focus and emotional orientation when confronted with new large-scale infectious diseases becomes crucial.

Research has shown that effective information dissemination can alleviate people’s fear of infectious diseases. Consequently, public awareness and an understanding of large-scale infectious diseases play a pivotal role in alleviating panic and prompting individuals to take action against this challenge. Personal perceptions of risk are often influenced by emotions. Positive emotions can make people more attentive and inclined to take proactive protective measures. When individuals have a more positive attitude toward pandemics, the recovery rate and control tend to be higher [1]. Conversely, negative emotions may lead to avoidance or inaction. Therefore, discussing the public’s attention to outbreaks of contagious diseases and the emotional shifts after the COVID-19 pandemic not only provides insights into changes in public attention to contagious diseases but also helps identify positive and negative emotions, as well as provides a more comprehensive understanding of the public’s stance on large-scale infectious diseases.

Simultaneously, discussing the public’s focus on large-scale infectious diseases and emotional orientation can not only help clarify the public’s perspectives on this issue but also assist in identifying the factors influencing emotions, both positive and negative, toward infectious diseases. Social media platforms play a vital role in disseminating information and shaping public opinion. Governments, health organizations, and public intellectuals can use these platforms to convey accurate information, reduce the spread of false information, and actively engage the public’s attention and actions regarding large-scale infectious diseases.

People are now more willing to express their opinions web-based and there is an abundance of data on social media platforms. Because of the convergence of opinions on the web, researchers can explore the changes in public discussion during the time change and likewise can focus on the public’s changing emotions about it. Considering current trends in technology, especially the role of computer science, it must be acknowledged that computer technology has made a major contribution to medical decision-making with regard to, for example, infectious diseases and epidemics [2,3]. The accurate and logical access sources of these data include social media platforms, which provide more valuable data than ever.

Weibo is among the largest and most influential social media sites in China. Weibo users can share their opinions, discuss current events, and express their emotions via PC using text, share pictures, and upload videos. Therefore, Weibo is an ideal platform for obtaining data sources of popular opinion texts. In addition, the opinions expressed on social networks are highly emotionally oriented; therefore, it is essential to analyze the emotions in the texts and content posted by users. Positive emotions are critical for motivation, perseverance, and prosocial behavior [4,5].

The existing literature on influenza sentiment orientation is mainly about COVID-19; for example, the study by Yin et al [6] is based on 13 million posts related to COVID-19 pneumonia collected over 2 weeks on Twitter (subsequently rebranded X), and the study by Harba et al [7] investigated how consumer sentiment evolved during the COVID-19 outbreak through content analysis and sentiment analysis of the texts of web-based restaurant reviews. Other mass infectious diseases have been studied to a lesser extent. Ng et al [8] studied public sentiment on the global outbreak of monkeypox on Twitter and analyzed 352,182 posts via unsupervised machine learning.

However, it is rare for an analysis of emotional orientation to analyze people’s attitudes toward other mass infectious diseases after experiencing the COVID-19 pandemic. Therefore, analyzing the public’s sentiment and changing views on the currently prevalent mass infectious disease, influenza A, through content posted on Weibo can accurately reflect the importance of public opinion in promoting policies related to epidemic prevention, increasing public awareness and participation in protective actions against the epidemic, and advancing the epidemic management process.

In surveys about emotions (questionnaires or interviews), respondents or interviewees may be influenced by the content of the questions or consider privacy issues and negative impacts, leading to difficulties in assessing emotions accurately and reasonably. Moreover, questionnaires do not allow access to, say, real-time influenza A sentiment, and data collection takes a long time and has high economic costs [9]. Therefore, we chose text mining as the research method to ensure the spatial and temporal diversity of data. Moreover, text mining applications have been used in various areas, including tourism [10,11], business [12,13], education [14,15], and health care [16-18] for a variety of beneficial purposes.

### Objectives

On the basis of the analysis described in the previous subsection, this study used a web crawler approach to obtain people’s opinions about influenza A. Thematic model analysis and sentiment analysis were used to explore people’s attention, concerns, and sentiments about the recent epidemic of the mass infectious disease. The topic analysis used latent Dirichlet allocation (LDA) to extract latent topics from comment text data. For comment text sentiment analysis, deep learning, that is, the bidirectional long short-term memory (BiLSTM) model, was chosen to classify sentiment. This study attempts to answer the following questions:

1. How concerned are people about the recently prevalent infectious disease, influenza A, after experiencing the COVID-19 pandemic? How does the concern differ from province to province?
2. What are the spatiotemporal differences between the total number of blog posts and the public’s attention to influenza A?
3. What are the changes in the public’s attitude toward infectious diseases after experiencing the COVID-19 outbreak? What are the most critical concerns of the people when a new infectious disease is spreading?
4. What is the public sentiment toward epidemic infectious diseases? How does it vary by region?
5. What are the drivers of positive and negative emotions?

## Methods

**Figure 1:**
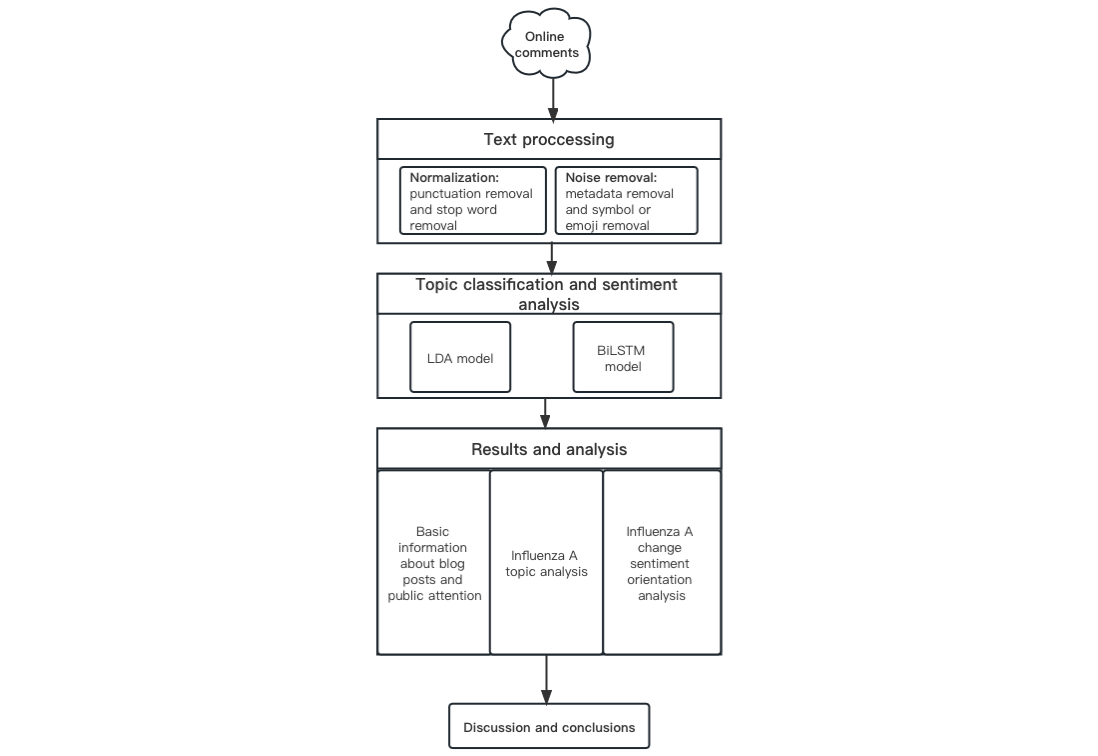
A schematic framework of the text data analysis method. BiLSTM: bidirectional long short-term memory; LDA: latent Dirichlet allocation.

We primarily used web crawling techniques to acquire data. After preprocessing the data, we further explored and analyzed the data using 2 models, LDA and BiLSTM, and obtained some meaningful conclusions, as shown in Figure 1.

### Preprocessing

We used web crawling techniques to collect 9351 posts on Weibo related to “influenza A” from November 1, 2022, to March 31, 2023. These data were used to create a data set that included user names, locations, posting times, content, repost counts, comments, likes, user types, and more.

To ensure the validity and stability of the data set, we removed duplicate data, deleted posts with <6 characters, and eliminated meaningless stop words, expressions, punctuation marks, and numbers. We also conducted semantic integration by summarizing words with similar meanings in the vocabulary.

### Text Mining Analysis

#### LDA Topic Model

**Figure 2:**
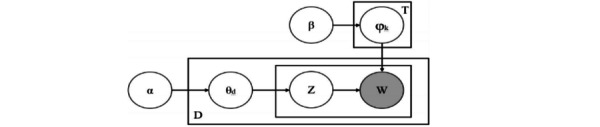
The latent Dirichlet allocation model generation process.

The standard topic models are latent semantic analysis, probabilistic latent semantic analysis, LDA, and hierarchical Dirichlet process. On the basis of text features and research needs, this study used LDA to extract latent topics from comment text data. LDA is an unsupervised machine learning technique that can identify potential topic information in large document sets or corpora. It uses a bag-of-words approach that treats each document as a vector of word frequencies, thereby converting textual information into numerical information that can be easily modeled. The model was first proposed by Blei et al [19] in 2003, along with the concepts and ideas of the LDA model. It is a 3-level Bayesian probabilistic model containing words, topics, and documents, and the document generation process is shown in Figure 2. In this study, the LDA model was used to investigate public attention to potential topics and understand the focus of public attention.

#### Sentiment Analysis

Comment text data are typically categorized into positive and negative sentiments for comment text sentiment analysis. There are 3 approaches to text sentiment analysis: sentiment analysis based on sentiment lexicon, sentiment analysis based on machine learning, and sentiment parsing based on deep knowledge. However, when it comes to sentiment analysis of medical service reviews, using a sentiment dictionary constructed based on electronic commerce reviews may lead to significant errors. Deep learning methods have shown clear advantages in sentiment analysis, breaking free from complex rule-based setups and demonstrating superior recognition performance, with the evaluation metrics and results significantly outperforming those achieved using traditional rule-based learning models. Research into deep learning models for sentiment recognition has primarily focused on the field of neural networks. However, owing to the large number of parameters in deep neural networks, they tend to overfit on limited data sets. To address this challenge, Vaswani et al [20] introduced the transformer deep learning model, which combines self-attention mechanisms, achieving fast and parallelized training and effectively addressing the issues of slow training and overfitting. Pretrained models have found extensive application in natural language processing tasks, particularly in domain-specific sentiment analysis. Nevertheless, regular corpora often fail to cover various domain-specific terminologies, resulting in certain limitations in the application of pretrained models such as bidirectional encoder representations from transformers in sentiment analysis research within the field of web-based sentiment. Research results indicate that, compared with other traditional sentiment analysis methods such as long short-term memory (LSTM), recurrent neural network, convolutional neural network, and naïve Bayes, BiLSTM models exhibit higher efficiency because they can effectively capture semantic information, achieving >90% accuracy in context understanding [21]. In sentiment analysis, positive and negative sentiments are typically the core focus because they directly relate to emotional polarity, which is crucial for many applications, such as sentiment trend analysis. Although some sentiment analysis tasks may include the classification of neutral sentiments, this choice often depends on specific application scenarios. Nevertheless, to maintain the research’s focus and clarity, we opted to solely concentrate on positive and negative sentiments. After careful consideration, we selected deep learning, specifically the BiLSTM model, for sentiment classification and categorized sentiment values into positive and negative emotions.

**Figure 3:**
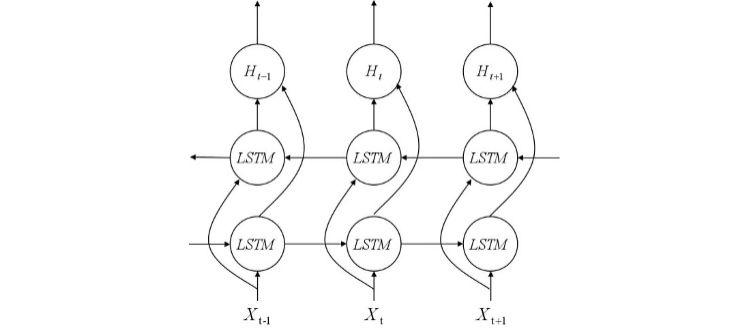
Bidirectional long short-term memory network structure. LSTM: long short-term memory.

**Figure 4:**
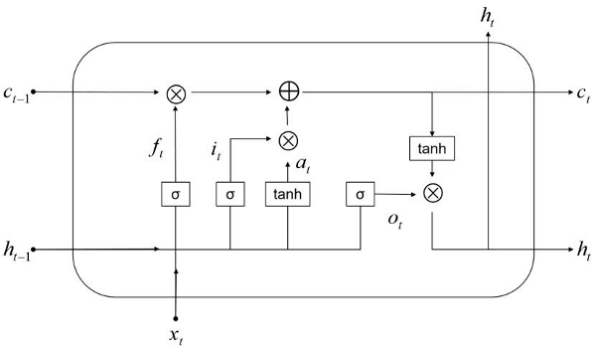
Internal structure of long short-term memory.

BiLSTM is a bidirectional recurrent neural network that takes the entire sentence’s words as input and considers the contextual information of the text. This allows information to be processed in both forward and backward directions [22,23]. As illustrated in Figure 3, BiLSTM combines forward LSTM and backward LSTM. Compared with convolutional neural network and LSTM, the BiLSTM model demonstrates superior performance, achieving an accuracy rate of >90% [21]. The internal structure of LSTM is depicted in Figure 4.

### Ethical Considerations

This study was approved by the medical ethics committee of the First People’s Hospital of Yunnan province (2022ZYFB001). The study used open-access social media data and excluded all personal information; therefore, informed consent was not required.

## Results

### Basic Information About Blog Posts and Public Attention

#### Trends in the Number of Blog Posts and Public Attention Over Time

**Figure 5:**
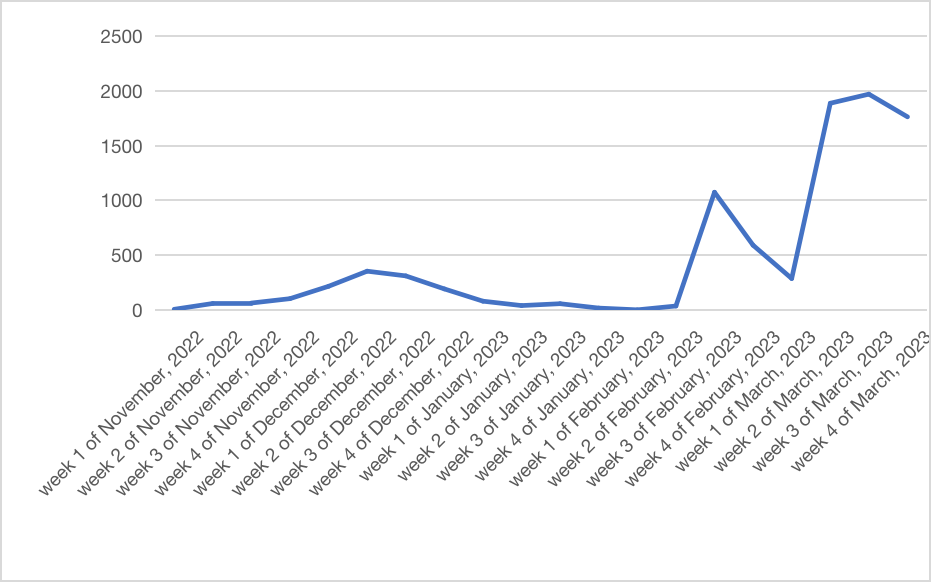
Time series of blogs about influenza A during the period from November 1, 2022, to March 31, 2023.

First, a fundamental descriptive statistical analysis of blog post volume was conducted to analyze the trend in public concern about influenza A. As seen in Figure 5, the public concern about the change in influenza A showed a significant increasing trend over time. In November 2022, there were 231 posts on Weibo related to the influenza A. In December, this number increased significantly to 1073 posts. Moving into January 2023, there were 194 posts, and in February, the number surged to 1703 posts. By March, the conversation intensified further, with a total of 5910 posts on the topic. After the COVID-19 pandemic, influenza A is a recent epidemic that has received much attention.

#### Spatial Difference Analysis of Blog Posts and Public Attention

There are considerable regional variations in the levels of concern about influenza A among Chinese provinces. This paper investigated the correlation between the number of blog posts and concerns about a potential mass epidemic in different areas of China, revealing some intriguing findings.

**Figure 6:**
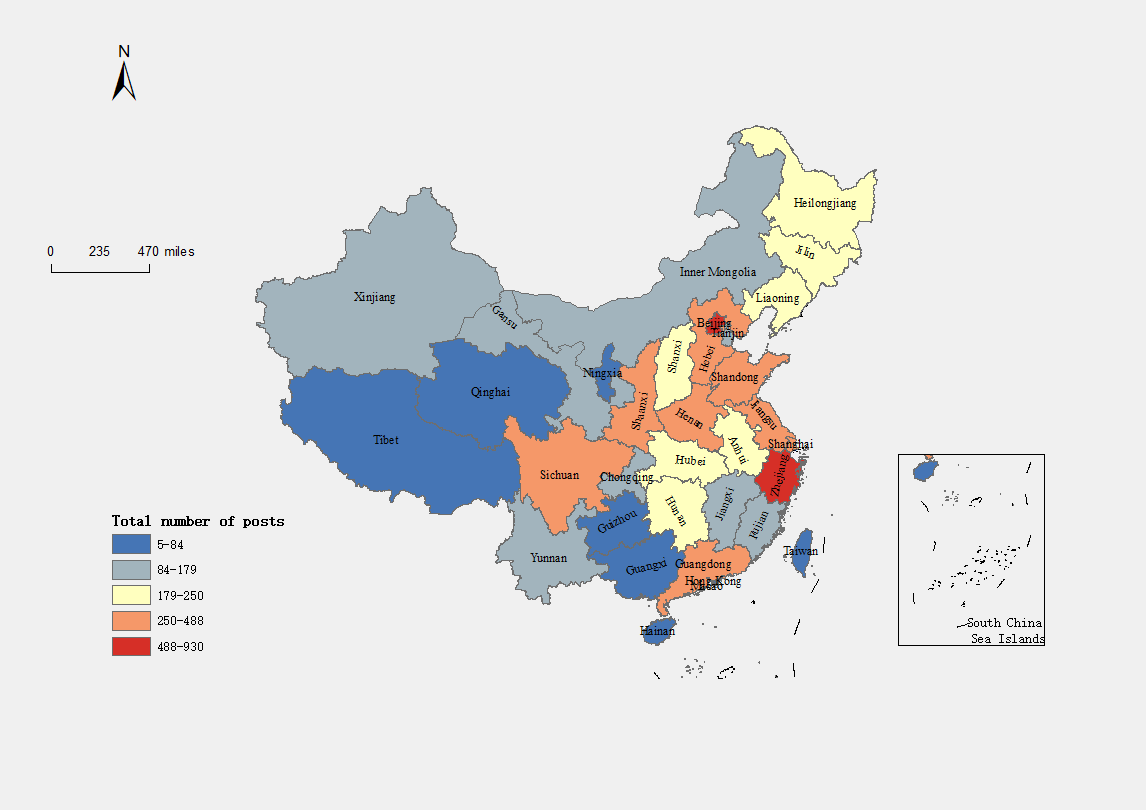
Number of blog posts and daily attention regarding influenza A in different provinces of China.

The study involved calculating the number of blog posts and the level of worry about influenza A for each of the 34 provinces. The findings, as depicted in Figure 6, highlight a substantial disparity in the number of blog posts between China’s eastern and western regions. Specifically, the Yellow River basin (including the provinces of Henan, Shandong, Hebei, and Shanxi) exhibits a relatively high number of blog posts, whereas the northwest region demonstrates the lowest. This pattern corresponds to the trapezoidal downward development trend observed in China, where the number of blog posts gradually diminishes from the eastern coastal areas to the western inland regions. Furthermore, the analysis identifies Beijing as the province with the highest number of published blog posts. In terms of ranking, Beijing, Zhejiang, Jiangsu, Shandong, and Sichuan occupy the top 5 positions. This indicates that these provinces expressed greater concern about influenza A than the others.

#### Comparative Analysis of Influenza A Attention Among Different Genders and User Types

Among different genders, there are 2122 male users and 5997 female users. It is evident that the number of posts made by female users surpasses that of male users. This observation suggests that women exhibit a higher level of concern about influenza A and actively engage in discussions on the internet regarding this topic. Their willingness to participate indicates a significant interest in the subject matter. Furthermore, Weibo classifies its users into 4 categories: blue V users, yellow V users, red V users, and regular users (the “V” label is akin to a verification symbol). Blue V users typically represent businesses or departments affiliated with certified institutions. These entities are required to undergo certification processes involving recognized organizations such as governments, businesses, schools, and media. Yellow V users, by contrast, are certified accounts belonging to renowned individuals in fields such as entertainment, sports, media, finance and economics, science and technology, literature and publishing, humanities and arts, games, military aviation, animation, tourism, and fashion, as well as government officials. Finally, red V users are certified accounts that achieve a minimum of 10 million monthly reads, granting them the red V certification. This distinction is a testament to the users’ popularity and influence on the platform.

Among all users, regular users make up the majority, accounting for 83% (7562/9111); following them are yellow V users at 10% (911/9111); blue V users at 5% (455/9111); while red V users comprise only 2% (183/9111). This observation implies that the topic of influenza A holds significant importance and captures the interest of the general public. The fact that ordinary users, who represent the majority of users on Weibo, display the greatest interest in this topic further emphasizes its relevance and the widespread concern among the public. It suggests that discussions and information related to influenza A are highly valued and sought after by ordinary individuals, highlighting the significance of this topic in the public discourse.

### Influenza A Topic Analysis

#### Word Frequency Analysis

The word frequency analysis, shown in Textbox 1, is used to analyze the concerns that people have about influenza A. The textbox shows that the words “Covid-19,” “infection,” “virus,” “influenza,” “flu,” and “feeling” are the main focus of people’s attention. This indicates that people will compare influenza, fever, and COVID-19 when concerned about influenza A and that the symptoms after falling sick are the most important. In addition to the aforementioned words, words such as “hospital,” “mask,” and “vaccine” appear more frequently. This indicates that people are also very worried about the related protective and treatment measures and the distribution of medical resources when concerned about influenza A.

**Result for the word frequency analysis.**

- Covid-19: 4445
- Infection: 3437
- Virus: 2946
- Influenza: 2447
- Fever: 2107
- Symptoms: 1843
- Hospitals: 1537
- Schools: 1324
- Flu: 1356
- Student: 1324
- Children: 1273
- Vaccine: 1206
- Outbreak: 1122
- Feeling: 1003
- Mask: 955
- Health: 904

#### Hot Topic Analysis

In this study, the LDA topic model was used for topic mining. The hyperparameters α and β were set as symmetric Dirichlet priors with values of 50/T and .01, respectively. The number of iterations for Gibbs sampling was set to 100, and the document contribution threshold ε was set to 1/k. The LDA model plays a crucial role in determining the number of potential topics and assigning meaningful labels to these topics. We used perplexity values to identify the optimal number of topics for the LDA model.

**Figure 7:**
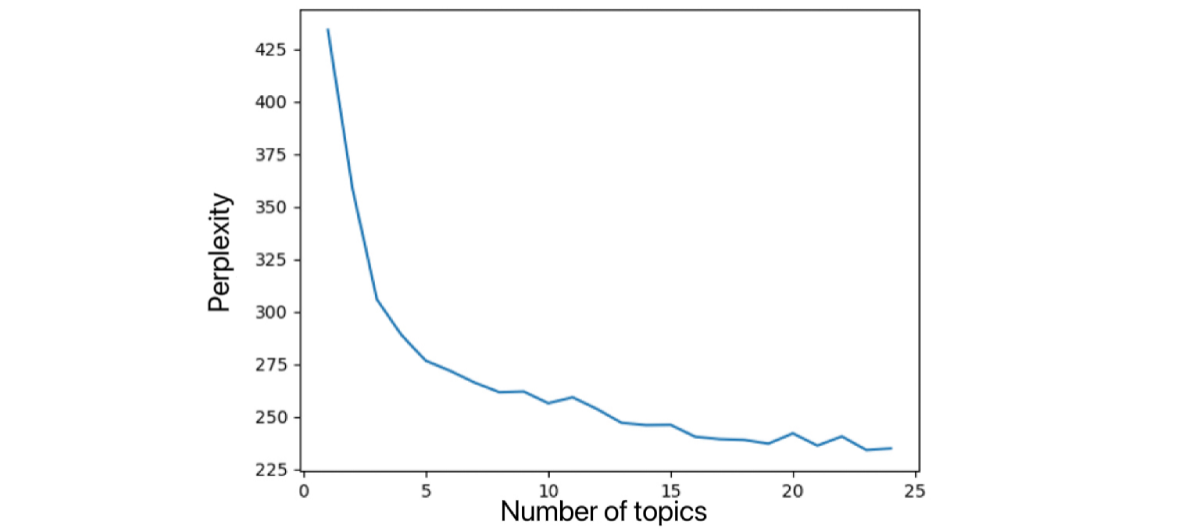
The result of perplexity value evaluation.

**Figure 8:**
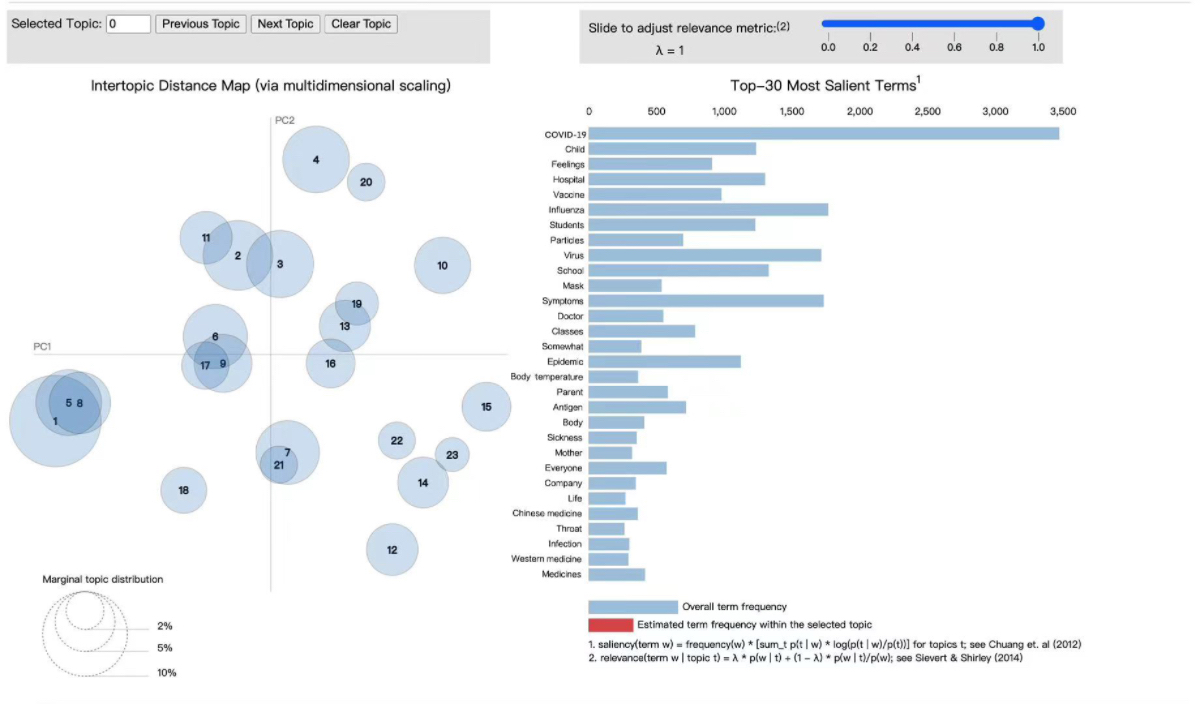
The results of the latent Dirichlet allocation (LDA) model (refer to Textbox 2 for the list of topics and keywords).

To determine the optimal number of topics, we conducted experiments with topic values ranging from 1 to 25 and generated a consistency curve fit, as depicted in Figure 7. On the basis of the results, 23 topics were identified as the most suitable for analysis. The right-hand section of Figure 8 illustrates the top 30 words with the highest frequency associated with each of the 23 topics.

From Figure 8, it is evident that there is a high degree of overlap among topics 1, 5, and 8. Textbox 2 reveals that these topics share common keywords such as “school,” “students,” and “classes.” The topics discussed revolve around the suspension of classes at primary and secondary schools owing to a rise in influenza A cases. In addition, topics 7 and 21 also exhibit a significant overlap. Moreover, there is a substantial crossover between topics 7 and 21, as well as among topics 6, 9, and 17. In Figure 8, the larger the circle in the left-hand section, the more critical the topic. Therefore, the blog posts with the highest attention paid to influenza A are topics 1, 2, 3, 4, 6, and 7. These highly discussed topics will be further analyzed.

**Topics and keywords (latent Dirichlet allocation).**

Topics and content

1. Covid-19, school, students, class, prevention, control, outbreak, command, primary school, virus, symptoms, children, testing, antigens, and precautions
2. granules, Pfizer medicine, western medicine, clinical, price, efficacy, Covid-19, national, Pfizer, pharmaceuticals, pharmacy, treatment, protocol, drug, and patients
3. pneumonia, epidemic, patient, patients, research, immunity, disease, symptoms, virus, situation, capsules, clinical, methods, traditional Chinese medicine, and time
4. influenza, symptoms, influenza virus, oseltamivir, drugs, antiviral, population, general, high Incidence, virus, dosing, patients, video, taking, and influenza vaccine
5. parents, students, Covid-19, positive, children, symptoms, virus, antigen, news, outbreak, school, class, test, virus infection, and elementary school
6. virus, influenza, Covid-19, human, transmission, everyone, virus strain, medical, nucleic acid, variant, positive, data, avian influenza, general, and mortality
7. company, pharmaceuticals, vaccines, limited company, pharmaceutical industry， national, work, Chinese Yuan, center, products, market, projects, production, sales, and hospitals
8. school, student, Covid-19, class, influenza, infectious disease, outbreak, primary school, symptoms, education bureau, situation, part, oral disease, and parents
9. vaccine, Covid-19, link, web page, biological, cell, protein, population, antibody, virus, variant, level, elderly, research, and antigen
10. feeling, throat, symptoms, sore throat, runny nose, slight, headache, body, body aches all over, stuffy nose, snot, whole body, pharynx, dizziness, and taste
11. hospital, viral, infection, influenza, patients, reporters, symptoms, disease, people, virus, situation, feelings, antigen, Covid-19, people’s daily, and nausea
12. sickness, infection, home, friends, teacher, classmates, school, exam, record, dormitory, colleague, good night, infection to, and almost
13. hospital, doctor, nucleic acid, test, check, outpatient, home, negative, symptoms, oseltamivir, community, queue, Covid-19 reinfection, daughter, and influenza
14. mom, son, dad, sister, adult, world, child, brother, life, diarrhea, family members, school, family, medicine, and housemate
15. body temperature, a little bit, all over the body, mental, state, bed, oseltamivir, hour, special medicine, antipyretic, day, affect, appetite, weakness, and aunt
16. child, fever medicine, hour, cooling, wave influenza A, All, warm water, disinfection, temperature, physical, everyone, situation, parents, moisture, and children
17. mask, month, personnel, ventilation, work, Covid-19, subtype, home, everyone, mobile, personal, time, first wave, and diligent hand washing
18. Covid-19, video, influenza B, school, aftermath, news, experiences, experts, positive rate, prevention, help, national, infectiousness, large number, and events
19. body, people, antigen, race, licorice, cold medicine, problem, situation, outrageous, advice, acute, magic, medicine, weight, ingredients, and pharyngitis
20. symptoms, soreness, general, advice, whole body, food nourishment, muscle, nasal congestion, infection period, inflammation, healthy, sore throat, throat, runny, and stomach
21. start school, infectious disease, basic, epidemic, family, spread, awareness, times, infectiousness, unable, probability, eyes, task, period, and science
22. life, kids, roommates, resistance, exercise, experts, parent, programs, moms, professors, gym, nutrition, dad, chief, physician, good, and news
23. kids, colleague, throat, influenza, vaccine, nose, infusion, go out, play, leader, thing, diary, neighborhood, oseltamivir, cake, care, and what

In topic 1, the keywords include “Covid-19,” “school,” “students,” “class,” “prevention and control,” “outbreak,” “command,” “primary school,” “virus,” “symptoms,” “children,” and “testing.” This topic focuses on the outbreak of influenza A in primary and secondary schools. Because of the gathering of people and the relative vulnerability of children, who are more susceptible to influenza A than adults, there is heightened societal concern about large-scale infections.

On social media platforms, individuals often express their concerns when their children contract influenza A. Parents who have not been infected themselves are worried about what preventive measures they can take against influenza A. These prevention efforts encompass a range of measures, including enhancing hygiene management and supervision at educational institutions; implementing disinfection and ventilation protocols; promptly identifying and isolating patients who have fallen ill and providing necessary treatment; raising awareness about protection measures among teachers, students, and parents; and reinforcing virus and antibody testing. Overall, the outbreak of influenza A at primary and secondary schools poses a significant public health challenge that necessitates collaborative efforts from the government, schools, parents, and the community to prevent and control its spread. Given the impact of the COVID-19 pandemic, there is heightened societal concern regarding mass infectious diseases, emphasizing the need for increased attention toward prevention and response to safeguard the health and safety of our children.

Compared with topic 1, topic 2 places more emphasis on the drugs used for combating influenza A, including their prices, efficacy, and the pharmaceutical manufacturers involved. Keywords associated with this topic include “Chinese medicine,” “Western medicine,” “clinical,” “treatment,” “Pfizer,” and “pharmacy.” The scarcity of drugs during the COVID-19 pandemic made it imperative to focus on drug-related aspects when addressing a new large-scale epidemic. Simultaneously, people want the pharmaceutical industry to develop specific drugs for contagious diseases, aiming to help individuals avoid illness. As is evident from the keywords, traditional Chinese medicine (TCM) holds a significant role in addressing the recent outbreaks of contagious diseases, garnering appreciation from the public. However, the issue of antibiotic misuse persists, particularly among patients with respiratory infections. Although various studies have been conducted to address the reduction of irrational antibiotic use, only a few have been multicenter or randomized trials. Exploring novel and innovative methods of administering medications is crucial to achieving the societal objectives of reducing irrational antibiotic use and eliminating unreasonable drug use. Therefore, providing education on the appropriate use of antibiotics during large-scale epidemic outbreaks is critical. Moreover, attention should be directed toward the drugs used to treat influenza A and their pricing to ensure that the public can access effective treatment promptly. This focus also aims to promote the research, development, and production efforts of pharmaceutical manufacturers in this field.

As depicted in Figure 8, topic 3 exhibits overlap with topic 2, sharing common areas of focus such as clinical aspects, methodologies, and TCM. Topic 3 specifically concentrates on the rational use of medications for managing influenza A symptoms. In light of the recent outbreak of the novel coronavirus, there has been heightened interest in mass infectious diseases, leading to a deeper understanding of the influenza A virus. This includes comprehending the symptoms caused by the virus, the human immune system’s response, and making comparisons with the novel coronavirus. Furthermore, individuals are likely to express concerns regarding the transmission of the influenza A virus, the efficacy of herbal treatments, and available clinical treatment options. Consequently, the outbreak of the novel coronavirus has significantly elevated the public’s awareness and comprehension of epidemic infectious diseases.

The topic 4 keywords encompass “influenza,” “symptoms,” “influenza virus,” “oseltamivir “ “drugs,” “antiviral,” “population,” “general,” “high incidence,” “virus,” “dosing,” “patients,” “video,” “taking,” and “influenza vaccine,” highlighting the focus on influenza A itself. Simultaneously with rapid ecological changes, accelerated urbanization, the impact of influenza A, and increased risks associated with travel and globalization, epidemics are becoming more frequent, complex, and challenging to prevent and control. In recent years, the general public has become increasingly aware of the health implications of epidemics, as evidenced by the appearance of keywords such as “antiviral,” “high incidence,” and “influenza vaccine” in topic 4. In conclusion, effectively responding to large-scale infectious diseases such as influenza A necessitates collaborative efforts among the government, medical institutions, pharmaceutical manufacturers, academia, and the public. By enhancing public education, improving preventive measures, and promoting rational drug use, the incidence and transmission risks of epidemics can be reduced, thereby ensuring public health and safety. In addition, it is crucial to learn from the experiences of the COVID-19 pandemic, enhance the public’s awareness and understanding of mass infectious diseases, and drive continual improvement and progress in epidemic prevention and control.

Keywords for topic 6 include “virus,” “influenza,” “Covid-19,” “human,” “transmission,” “everyone,” “viral strain,” “medical,” “nucleic acid,” “variant,” “positive,” “data,” “avian influenza,” “general,” and “mortality.” The focus is on the discussion of viruses. Influenza A is an influenza virus that belongs to the family Orthomyxoviridae, a different family of viruses than the novel coronavirus. The virulence of the influenza A virus is relatively low, but it spreads quickly and is easily disseminated among the population. The main symptoms of influenza A include fever, cough, sore throat, muscle pain, fatigue, and headache, which usually appear within 2 to 3 days after infection. The mortality rate of the influenza A virus is low. However, it may cause more severe complications in specific populations, such as older adults, young children, pregnant women, and people with weakened immune systems. It is important to note that the influenza A virus and the novel coronavirus have different characteristics and impacts on public health. It is worth noting that topic 6 mentions comparisons with previous major infectious viruses when discussing influenza A viruses, including the ones responsible for COVID-19 and avian influenza.

Under topic 7, the keywords include “company,” “pharmaceuticals,” “vaccines,” “limited company,” “pharmaceutical industry,” “national,” “work,” “Chinese Yuan” “center,” “products,” “market,” and “projects.” This topic focuses on the public’s interest in pandemic vaccines. Influenza viruses are classified into 3 serotypes: A, B, and C. Type A has the potential to cause large-scale epidemics owing to the variation in the structure of its antigens, which occurs approximately once every 10 to 15 years. Type B epidemics are typically milder and more limited in scope, whereas type C generally causes milder epidemics. Humans are universally susceptible to all 3 types, and all 3 types can cause various respiratory conditions such as laryngitis, bronchitis, bronchiectasis, capillary bronchitis, and pneumonia.

**Figure 9:**
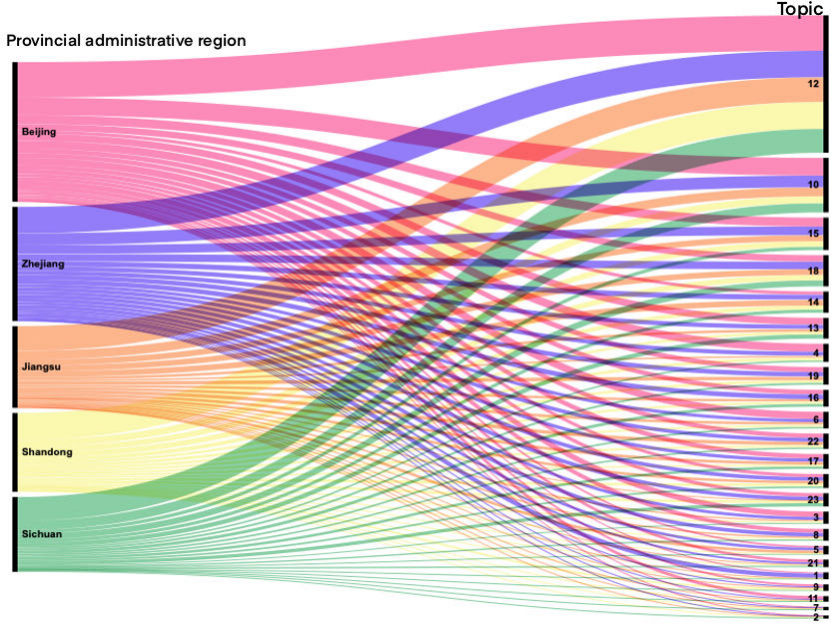
Analysis of the proportion of different topics with regard to influenza A in Beijing, Zhejiang, Jiangsu, Shandong, and Sichuan (refer to Textbox 2 for the list of topics and keywords).

In Figure 9, the left-hand side represents the 4 provinces with the highest posting activity, whereas the right-hand side shows the number of posts corresponding to negative emotional themes. The research findings indicate that the topic of greatest concern among users is topic 12, which revolves around infections in schools. This is primarily because of the closure of schools after the influenza A outbreak, and the susceptibility of children in school environments to infection. The next topic of interest is topic 10, which includes keywords such as “feeling,” “throat,” “symptoms,” “sore throat,” “runny nose,” “slight headache,” “body aches over all,” “stuffy nose,” “snot,” “whole body,” “dizziness,” and “taste.” This topic pertains to postinfection symptoms because the symptoms associated with influenza A infections are prominent, leading individuals to experience physical and emotional distress, thereby contributing to more negative sentiment.

### Influenza A Change Sentiment Orientation Analysis

#### Spatial Difference Analysis

**Figure 10:**
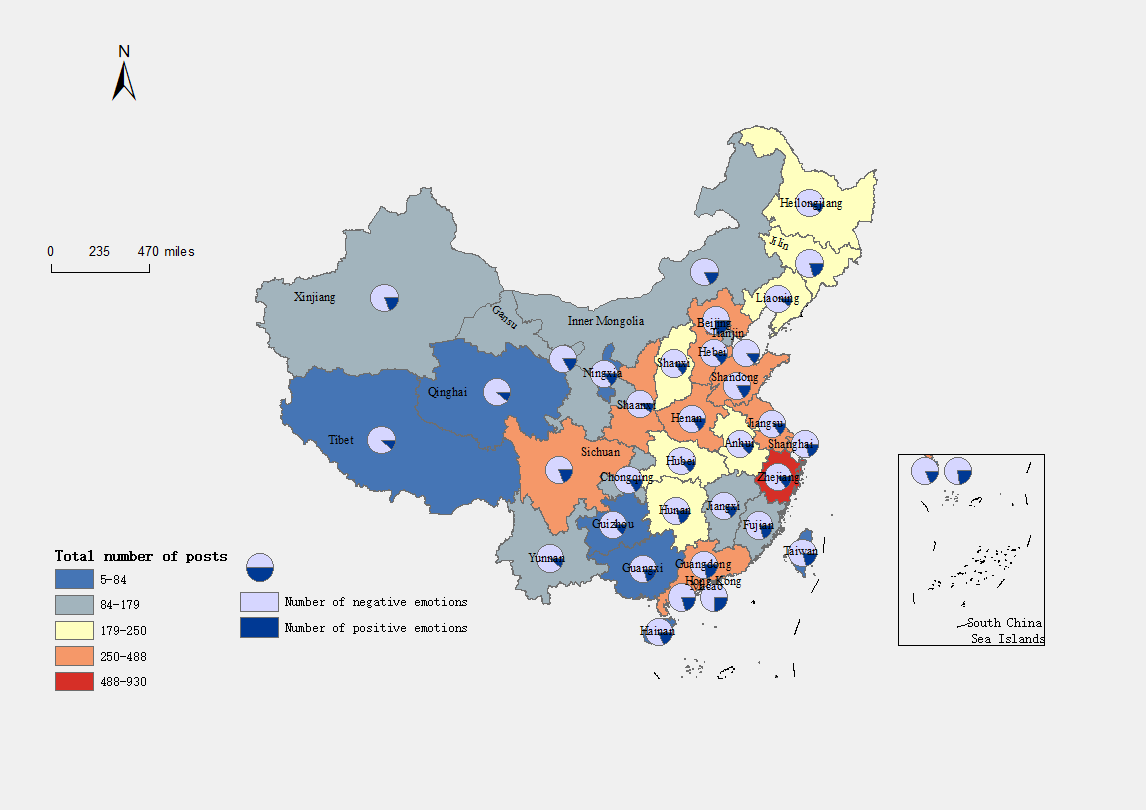
Number of blog posts and daily attention regarding influenza A in different provinces. The pie charts show the distribution of sentiment orientation in each area.

Emotional distribution can reflect the public’s attitude and sentiment toward relevant issues. In this paper, we categorized emotional orientation as positive or negative. According to our analysis, the public’s emotional exposure toward influenza is mainly negative, with negative emotions accounting for 83% (7562/9111), whereas positive emotions account for only 17% (1549/9111). This indicates that although the COVID-19 pandemic has brought many adverse effects, the public’s attitude toward influenza still needs to be more optimistic. We also analyzed the emotional orientation of different provinces toward influenza A. Figure 10 shows that each region holds a negative attitude toward influenza A, and there is little difference in the ratio of positive and negative emotions. We mainly focused on 3 regions—Qinghai, Yunnan, and Tibet—and found that they have a stronger negative emotional orientation than other sites.

#### The Factors Influencing Sentiment Orientation

On the basis of the study’s analysis, further exploration was conducted to understand the reasons behind positive and negative emotions among the public regarding influenza A. In the word frequency analysis concerning positive emotions, the following terms hold significance within the data set: “COVID-19” appears 1863 times, “influenza A” is documented 1768 times, “Infection” occurs 1596 times, “Virus” is mentioned 1033 times, “Influenza” is noted 1008 times, “Symptoms” is found 1001 times, “Control” is used 909 times, “Prevention” appears 854 times, “Outbreak” is mentioned 829 times, “Vaccine” occurs 818 times, “Fever” is referenced 719 times, “Children” is included 709 times, “Hospital” appears 706 times, “Malaise” is used 681 times, and “Classes” is seen 622 times. In addition to the high-frequency term “influenza A,” the public often discussed terms such as “prevention,” “control,” and “vaccine,” an indication of their concern and focus on influenza A. This suggests that the positive sentiment toward the influenza A epidemic primarily stems from effective prevention and control measures and the availability of a reliable vaccine. Furthermore, discussions about antiviral drugs and treatment reflect the public’s trust and expectation of scientific treatment options.

In the word frequency analysis pertaining to negative emotions, the following terms play a significant role in the data: “influenza A” appears 6069 times, “COVID-19” occurs 1729 times, “fever” is found 1373 times, “infection” appears 903 times, “myself” is present 820 times, “today” is mentioned 816 times, “influenza” is documented 778 times, “symptoms” is noted 759 times, “cold” is mentioned 730 times, “feeling” appears 721 times, “virus” occurs 679 times, “hospital” is seen 616 times, “uncomfortable” is used 599 times, and “child” is included 527 times. Finally, “cough” is listed 522 times. The common words associated with negative sentiment in blog posts, including “infection,” “fever,” and “symptoms” reflect the negative emotions stemming from public concern about the influenza A outbreak and the discomfort experienced by those who fall sick. In addition, inaccurate rumors and misunderstandings can contribute to negative emotions among the public. Therefore, it is crucial to disseminate scientific and accurate information while implementing timely epidemic prevention and control measures. These actions can effectively alleviate negative emotions, enhance public confidence and resilience, and collectively address the challenges posed by the influenza A epidemic.

## Discussion

### Principal Findings

The level of concern regarding the recently prevalent infectious disease, influenza A, has shown variations across Chinese provinces, influenced by the experience of the COVID-19 pandemic. Notably, the central region of China seems to display a heightened level of concern, whereas the northwest region exhibits a lower level of attention. This geographic disparity is reflected in both the total number of blog posts and the public’s attention to influenza A, demonstrating fluctuations over time. These fluctuations underscore the dynamic nature of public attention to infectious diseases and emphasize the necessity for region-specific communication strategies. Furthermore, the research findings suggest that individuals become more sensitized with regard to infectious diseases and exhibit increased levels of concern, especially in the face of the spread of a new infectious disease.

### Interpretation

#### Spatiotemporal Differences Between Blog Posts and Public Attention

From November 1, 2022, to March 31, 2023, there was an increase in the number of posts related to influenza A, indicating a growing concern among the public regarding this issue. Figure 6 illustrates that the number of posts is relatively lower in the western region and higher in the eastern part of the country, with a concentration in the central area. This pattern may be attributed to the higher population density and greater mobility in the eastern part, leading to a faster spread of influenza A and prompting more people to pay attention to the topic and discuss it. In addition, the central region, characterized by a more densely populated area, facilitates frequent information exchange among its residents, resulting in an increased number of posts on Weibo. Notably, Beijing has the highest number of posts among all provinces. This can be attributed to Beijing being a region with high population density and significant mobility, which may contribute to a faster spread of influenza A and generate more attention and discussion on the topic. Moreover, Beijing’s advanced internet infrastructure and the widespread adoption of social media platforms also contribute to the higher number of posts.

#### Difference Analysis of Influenza A Attention Among Different Genders and User Types

This study of the genders and types of users shows that female users are much more concerned about influenza A than men. This can be explained in several ways. First, women are more concerned about health and personal hygiene issues [24], which makes them more worried about the influenza A outbreak. Second, more women than men work in medical and nursing professions [25], which means that diseases such as influenza A are top of mind for them. This also contributes to their higher level of concern about influenza A. In addition, information about influenza A is usually more widely disseminated by women in the family [26]. Women typically play more active roles in the family as primary family caregivers, guardians of children, and so on [27]. Therefore, they are more likely to spread information about influenza A within the family. In addition, some studies show that women are better at expressing emotions and empathy [28]. Women’s risk perception ability is sharper when faced with a public health event [29], and they are more likely to pay attention to information about influenza A. From another perspective, there is a reason why there are many Weibo users with posts related to influenza A. First, influenza A is a prevalent infectious disease that can affect most people. Therefore, many people are concerned about information related to influenza A. Second, the symptoms of influenza A are similar to those of some common diseases, such as cold and influenza, which makes many people search for influenza A–related information when they have similar symptoms.

#### Hot Topic Analysis

Building from previous studies that focus on influenza [30-32], this study highlights that the health topic of greatest public concern in China is influenza A and its characteristics. As a highly contagious disease, influenza A, which shares similarities with influenza, is known to be more painful than influenza and prone to severe complications, including death. Consequently, the public is eager to acquire more information about influenza A to safeguard their health and that of their families. Furthermore, both the novel coronavirus and the influenza A virus are respiratory viruses, prompting comparisons between the two. Consequently, understanding the differences and similarities between influenza A and COVID-19 can empower the public to comprehend both diseases better and adopt more effective preventive and control measures.

The next topic of interest is viruses. As influenza A is a virus-transmitted disease, it is essential to understand its virulence, symptoms, mortality rate, and transmission rate to understand the disease. This is also because the spread of the virus directly affects public health and social stability; therefore, naturally, the public is concerned about the virus.

Another topic is the public’s concern about preventing mass epidemic infectious diseases through the use of vaccines. The reasons for this are easy to understand. First, as vaccines are one of the most effective measures to prevent disease [33,34], the public began to pay more attention to the development of a vaccine in the hope that reliable preventive measures would be available early. Considering the role played by vaccines during the COVID-19 pandemic, we can see the importance of vaccines in controlling the spread of diseases and providing the public with effective measures to prevent the spread of epidemics. Second, public concern is also related to the safety and efficacy of vaccines [35,36] because vaccinating oneself is a significant decision involving everyone’s health and life. Finally, the public’s concern about epidemic vaccines is also related to health care systems and policies [36,37]. Vaccine development, production, and distribution require the support and regulation of health care systems and policies.

In addition to prevention, people are also concerned about the drugs used to treat influenza A, the price and efficacy of the drugs, and the drug manufacturers. This may be related to the COVID-19 outbreak. As the COVID-19 outbreak continues to pose a threat to people’s physical and mental health, there is still concern among the public about contracting the virus. In addition, people are also worried about the efficacy and side effects of antiviral medications and want to know details about their safety and applicability to make the proper treatment choice [38]. Pharmaceutical manufacturers have also become the focus of public attention because they are essential players in producing influenza A treatment drugs. Many TCM institutions and physicians actively responded during the COVID-19 pandemic and achieved some significant treatment results [39-41]. This also drew public attention to TCM’s role during the epidemic, and people increasingly value TCM; in fact, the treatment of influenza A by TCM has received much attention [42-45].

The next concern is the rational use of medication after contracting influenza A. This may be related to the COVID-19 outbreak, in the sense that the public is more concerned now about using the correct medications to relieve influenza A symptoms. In this context, the public is more concerned about using medications to relieve influenza A symptoms correctly. In addition, owing to the popularity of the internet and social media, public health awareness is gradually increasing, and people are more willing now to actively seek health information and treatment advice [46-48]. At the same time, the continuous advancement of medical technology has made the treatment methods for influenza A more and more diversified and precise, making the public more concerned about the rational use of medication to treat influenza A.

One fascinating topic was the influenza A outbreak in primary and secondary schools. This relates to the closure of primary and secondary schools in China during the COVID-19 pandemic when the government took several measures to prevent the spread of the disease. This resulted in students being unable to attend school, and many students began to study independently or receive distance learning at home. This situation has led to an increase in parents’ concerns about the safety and hygiene standards prevalent in schools and other educational institutions [49-51]. Besides, it is known that schools can become a source of mass infections among children.

#### Sentiment Orientation Analysis

Understanding public sentiment regarding the influenza A epidemic in light of the COVID-19 outbreak is crucial because it reflects public perceptions and attitudes toward health and disease, as well as their level of confidence and trust in outbreak prevention and control measures. The results of our study indicate that 83% (7357/9111) of Chinese individuals hold negative attitudes toward influenza A. These negative emotions are not primarily directed at the government or official institutions but rather stem from people’s psychological distress and anxiety regarding physical discomfort because influenza A can cause physical pain, fever, cough, and weakness, leading to individuals feeling unwell and physically burdened; in addition, given the recent experience of the COVID-19 pandemic, the emergence of influenza A exacerbates people’s psychological exhaustion and weariness.

At the same time, there are also positive emotions associated with confronting influenza A; for example, the efforts of the Chinese government in implementing various measures to address the influenza A outbreak, including vaccination programs, have helped the public to better cope with the outbreak and instilled confidence in the government’s response.

### Limitations

#### Data Source Limitations

It is important to acknowledge the limitations of our data source. Weibo users, primarily composed of the younger demographic, may not provide a comprehensive representation of society as a whole. Furthermore, the attitudes and sentiments expressed on Weibo may not be entirely reflective of the broader societal attitude. It is crucial to recognize that Weibo users’ opinions may not necessarily encompass the perspectives of the entire community.

#### Spatiotemporal Analysis Constraints

Our study’s spatiotemporal analysis is subject to certain constraints. Specifically, we focused on analyzing people’s attitudes toward influenza A during a specific time frame after the COVID-19 pandemic. Unfortunately, we did not conduct a comparative analysis of attitudes before and during the COVID-19 pandemic. This limitation restricts our ability to provide insights into how the pandemic might have influenced changes in attitudes over time. In future research, comparing prepandemic and pandemic-era attitudes could yield valuable additional insights.

### Conclusions

#### Overview

The COVID-19 pandemic has significantly increased public awareness of mass infections and the importance of preventive and control measures. In this context, this study on influenza A and its analysis of public sentiment provide valuable insights into the changing attitudes and concerns of the public. These findings can positively affect epidemic prevention and control efforts in the following ways.

#### Effective Communication Policies

Understanding public sentiment regarding the influenza A epidemic empowers the government and health organizations to devise communication policies tailored to the public’s perceptions and concerns. By addressing these, they can enhance the public’s comprehension of the outbreak and encourage the adoption of suitable protective measures. This proactive communication strategy plays a pivotal role in effectively curbing the spread of the epidemic; for instance, the government may implement a comprehensive communication plan, including daily updates on infection rates, guidelines for mask wearing, and information on vaccination centers, all designed to keep the public well informed.

#### Promoting Vigilance and Preventive Awareness

Positive public attitudes toward the influenza A epidemic can heighten public vigilance and awareness of preventive measures. A positive outlook encourages individuals to proactively engage in protective behaviors such as regular handwashing, consistent mask use, and avoidance of crowded areas. These actions reduce the risk of infection and contribute significantly to slowing down the transmission of the virus. To promote this, public health campaigns can emphasize the role of these behaviors in reducing transmission rates and saving lives.

#### Promptly Addressing Public Concerns

Understanding public attitudes and concerns about the influenza A epidemic equips health organizations and government authorities to promptly respond to public inquiries and address worries. By strengthening public information campaigns and educational initiatives focused on influenza A, they can bolster the public’s confidence and willingness to cooperate with recommended control and prevention measures; for example, they may establish hotlines or web-based forums where experts provide real-time answers to common questions, alleviating public concerns and building trust in official guidance.

#### Risk Assessment and Adaptive Policies

Negative public sentiment regarding influenza A in China indicates the necessity for a comprehensive risk assessment. Understanding public opinion allows health organizations and government entities to swiftly adapt prevention and control measures. This includes the development of targeted policies and guidelines that align with the evolving public sentiment; for instance, if negative sentiment arises owing to perceived vaccine shortages, authorities can swiftly adjust vaccine distribution strategies and communicate these changes transparently to rebuild trust.

#### Enhancing Management Capacity and Public Cooperation

A profound understanding of public sentiment helps enhance the management capacity of health organizations and government bodies. It strengthens communication channels and cooperation with the public, fostering a more robust social collaboration mechanism. This, in turn, facilitates the seamless implementation of epidemic prevention and control measures; for example, regular public engagement forums can be established, allowing citizens to voice concerns and provide input into decision-making processes, ultimately leading to more effective and inclusive policies.

By actively considering public sentiment, health organizations and the government can not only engage the public more effectively but also tailor their strategies and policies to better address the challenges presented by the influenza A epidemic.

## Abbreviations

BiLSTM: bidirectional long short-term memory
LDA: latent Dirichlet allocation
LSTM: long short-term memory
TCM: traditional Chinese medicine

## Data Availability

The data sets generated and analyzed during this study are available from the corresponding author on reasonable request.
